# Prophylactic Administration of Surfactant in Extremely Premature Infants

**DOI:** 10.1155/2010/235894

**Published:** 2010-06-07

**Authors:** Lutz Koch, David Frommhold, Bernd Beedgen, Peter Ruef, Johannes Poeschl

**Affiliations:** Division of Neonatology, Department of Pediatrics, University of Heidelberg Medical School, Im Neuenheimer Feld 430, 69120 Heidelberg, Germany

## Abstract

*Objective*. To investigate whether prophylactic surfactant administration is superior over selective treatment in preterm infants with respiratory distress syndrome (RDS). *Methods*. In our retrospective analysis, we compared premature infants (23 + 0 to 26 + 6 weeks) receiving 200 mg/kg surfactant (curosurf^®^) within five minutes after birth (prophylactic group, *N* = 31) with those infants who received surfactant therapy for established RDS (selective group, *N* = 34). *Results*. Prophylactic therapy significantly decreased the need for mechanical ventilation (74 hours per patient versus 171 hours per patient, resp.). We observed a reduced incidence of interstitial emphysema (0% versus 9%, resp.), pneumothoraces (3% versus 9%, resp.), chronic lung disease (26% versus 38%, resp.), and surfactant doses per patient (1.3 versus 1.8, resp.), although those variables did not reach significance. *Conclusion*. We conclude that infants under 27 weeks' gestation profit from prophylactic surfactant administration by reducing the time of mechanical ventilation. This in turn could contribute to reduce the risk for mechanical ventilation associated complications, without any detrimental short-term side effects.

## 1. Introduction

Evidence from randomized, controlled trials, as summarized in the Cochrane systematic reviews, demonstrates that prophylactic surfactant administration to infants judged to be “at risk” for developing respiratory distress syndrome, compared to selective use of surfactant in infants with established RDS, improved outcomes for high-risk preterm infants [1]. The use of surfactant for the treatment or prophylaxis of neonatal RDS results in a 30–65% relative reduction in the risk of pneumothorax and up to a 40% relative reduction in the risk of mortality. Adverse events are infrequent, and long-term follow-up studies are reassuring. Prophylactic administration of surfactant may be preferable to rescue treatment, especially in infants <30 weeks of gestation, as it decreases the risk of pneumothorax, pulmonary interstitial emphysema, and neonatal mortality [2], but may result in some babies being intubated and receiving therapy unnecessarily. These trials were carried out in the early 1990s when prenatal steroids were used less than currently and the respiratory assistance with noninvasive nasal continuous positive airway pressure (CPAP) was poorly adopted in very preterm infants. Nasal CPAP is currently a first-line technique of respiratory support in newborns, and its use as an alternative to intubation and mechanical ventilation, also in extremely low gestational age infants, is well documented [3, 4], but no clinical trial compared initial CPAP and rescue surfactant therapy with initial intubation and prophylactic surfactant administration. Therefore, it remains unclear which criteria should be used to select “at risk” infants who would require prophylactic surfactant administration.

In our retrospective analysis, we based our comparison of prophylactic with selective surfactant treatment on the hypothesis that infants under 27 weeks' gestation are “at risk” in developing RDS and may profit from prophylactic surfactant administration.

## 2. Methods

Preterm infants with a gestational age between 23 + 0 and 26 + 6 weeks + days born in our center during the observation period from January 2007 to December 2007 were eligible for the study. 

Infants included in the study received 200 mg*kg^−1^ of a natural surfactant preparation (curosurf^®^, Chiesi, Germany) via intratracheal tube within five minutes after birth. The outcome criteria were compared with a historical control group of patients treated in the 12 months immediately before the observational period. These control infants were treated with CPAP at 5-6 cm H_2_O and were intubated and received surfactant therapy only for established RDS FiO_2 _> 0.4 to maintain SpO_2_ between 85% and 93%). Infants who needed tracheal intubation and mechanical ventilation for primary resuscitation received surfactant within 20 minutes after birth. In both study groups, infants were extubated as soon as FiO_2_< 0.3 and a mean airway pressure <7cm H_2_O was reached. Immediately after extubation, CPAP at 5-6 cm H_2_O was started using a stephanie respirator. CPAP was discontinued when the neonate remained stable with PCO_2_, oxygen saturation, and no CPAP for more than four consecutive hours. No other changes in intensive care management occurred during the study period.

Outcome criteria such as chronic lung disease (CLD), defined as the need of additional oxygen after 36 weeks gestational age, intraventricular hemorrhage (IVH) > II according to the classification of Papile, periventricular leukomalacia (PVL), pulmonary interstitial emphysema (PIE), patient ductus arteriosus (PDA), rate of nosocomial infection, usage of postnatal steroids (hydrocortisone), and necrotizing enterocolitis (NEC) stadium II or III according to Bell are also reported.

Echocardiographic criteria for treatment of PDA included an increased left atrial diameter compared with aortic root (ratio> 1.2), visualization of the ductus (>2 mm), and evidence of left-to-right blood flow through the open duct as summarized by Sperandio et al. [5]. Nosocomial infection was defined according to NEO-KISS criteria. It is based on the US Centers for Disease Control and Prevention (CDC) criteria and has been adapted for this specific age group [6].

Postnatal steroid therapy with hydrocortisone was started when the postnatal age was >1 week and the children was ventilator-dependent with increasing oxygen requirements or needed oxygen >40% on CPAP or oxygen cannula. Infants received hydrocortisone according to a well established nine-day protocol: 3 days 45 mg/m^2^/d (~4-5 mg/kg), tapered to 30 mg/m^2^/d (~3 mg/kg) for another 3 days followed by 15 mg/m^2^/d (~1-2 mg/kg) for 3 days, given 3 times a day according to the circadian rhythm.

The study was carried out in accordance with ethical guidelines for human studies.

## 3. Statistics

Data are given as mean and range or number (percentage) and were analyzed with SPSS software (SPSS for Mac, version 16.0, Chicago, IL). The baseline characteristics of the two groups were compared using Student's *t*-test for parametric and the chi-squared test for nonparametric comparisons. *P*-values <.05 were considered statistically significant.

## 4. Results

Thirty-one infants with a gestational age between 23 + 0 and 26 + 6 weeks + days were born and treated in our center during the observational period of 12 months. There were no significant differences between birthweight, gestational age, sex, use of antenatal steroids, APGAR-score, rate of SGA (small for gestational age, birth weights below 10th percentile), and rate of multiple births of all patients treated in the observational period and historical control period as Table 1 shows. In each group, one patient suffered from serious hydrops fetalis. Twin to twin transfusion appeared in one twin birth in each group. Furthermore, in the selective surfactant group one patient had unknown trisomy 18.

Table 2 shows the mortality and morbidity rates for all patients of the prophylactic and selective surfactant group. Prophylactic surfactant therapy was associated with improved respiratory outcome. The duration of mechanical ventilation was tremendously and significantly shorter in the prophylactic surfactant group compared to the selective surfactant group (74 hours per patient versus 171 hours per patient, resp.). In addition, we found a reduced incidence of PIE (0% versus 9%, resp.), pneumothoraces (3% versus 9%, resp.), and CLD (26% versus 38%, resp.), although those variables did not reach significance. 

There were no differences between diagnosis (71% versus 82%, resp.) and treatment (68% versus 68%, resp.) of PDA and nosocomial infections (45% versus 44%, resp.). The incidence of NEC stadium II or III according to Bell was increased in the selective surfactant group (3% versus 15%, resp.), although this variable did not reach significance.

The use of postnatal steroids was not different in both groups (61% versus 62%, resp.). Interestingly, we found a decreased number of surfactant doses used per patient in the prophylactic group (1.3 versus 1.8, resp.).

## 5. Discussion

Recent randomized and controlled trials demonstrated that prophylactic or early surfactant as compared to delayed rescue surfactant treatment results in improved outcomes for high-risk preterm infants [1, 7, 8]. However, the trials did not provide a definitive answer to the question of best timing of surfactant application at different gestational ages. Moreover, these data have been obtained in the early 1990s when prenatal steroids were used less than currently and the respiratory assistance with noninvasive nasal CPAP was poorly adopted in very preterm infants. Despite the advantages of prophylactic surfactant strategy for infants born less than 30 weeks' gestation, many such infants treated only after RDS has become established. In a large North American cohort of 47608 infants less than 30 weeks of gestation born between 1998 and 2000, only 27% received surfactant in the delivery room and 44% received surfactant by 30 minutes of age. Even later, (before 6 hours of age) still 29% had been given surfactant [9]. In the group of infants with gestational age less than 27 weeks, only 32% received surfactant in the delivery room. The European consensus guidelines now recommend a prophylactic surfactant therapy within 15 minutes of birth to almost all babies under 27 weeks' gestation [10]. In our study, we address the question whether this strategy improves respiratory outcome in very premature infants under 27 weeks' gestation.

According to other studies [1, 7], we found a reduced incidence of pulmonary interstitial emphysema and pneumothoraces, although those variables did not reach significance. The risk of developing chronic lung disease after prophylactic surfactant compared with rescue surfactant has been discussed and remains controversial. Secondary analyses of clinical trials reported no different [1], decreased [11–13], and increased [14] risk of CLD in preterm infants who received prophylactic surfactant compared with those who received later selective surfactant treatment. Reininger et al. [15] compared the effect of one dose of intratracheally administered surfactant followed by extubation to nasal CPAP with nasal CPAP alone in infants 29 to 35 weeks' gestation with mild-to-moderate RDS requiring supplemental oxygen and NCPAP. 57% of the infants in the control group and 46% of the infants in the surfactant group received antenatal steroids. Reininger et al. showed a significantly decreased need for mechanical ventialtion with the use of prophylactic surfactant administration and identified a trend toward a decrease in the incidence of bronchopulmonary dysplasia (BPD). 

In line with these studies, we also observed a trend to a decreased incidence of chronic lung disease. Nevertheless, the significant shortening of the time of mechanical ventilation turned out to be the most dramatic result of our study. This in turn could contribute to reduce the risk for mechanical ventilation associated complications. 

Nasal CPAP is currently a first-line technique of respiratory support in newborns, and its use as an alternative to intubation and mechanical ventilation, also in extremely low gestational age infants, is well documented [3, 4]. Few randomized trials have compared different approaches of intubation with surfactant administration and mechanical ventilation versus early nasal CPAP. The results of these studies did not highlight significant differences [16–18]. Morley et al. [17] compared initial CPAP (8 cm H_2_O) with initial intubation in the delivery room in infants 25 to 28 weeks of gestational age (COIN trial). They found a better outcome at 28 days in the CPAP group compared to the intubation group; the two groups had a similar outcome at 36 weeks' gestational age. Early nasal CPAP did not significantly reduce the rate of death or bronchopulmonary dysplasia, as compared with intubation. The benefits of CPAP included a lower risk of combined outcome of death or the need for oxygen therapy at 28 days and fewer days of assisted ventilation. There is no information about the days of assisted ventilation in the CPAP subgroup of infants at 25 or 26 weeks' of gestation. In contrast, we found a significant decrease in the need for mechanical ventilation after intubation and prophylactic surfactant administration. In the COIN trial, sick infants who required immediate intubation were excluded and timing, dosing, and preparation of surfactant were not mandated and followed local protocols. The death rate at day 28 in the CPAP subgroup of infants at 25 or 26 weeks' of gestation was more than twice as high as in the intubation group (odds ratio 2.04; 95% CI 0.72–5.74), but this failed to meet statistical significance. 

Verder et al. [19, 20] showed a low mortality and low morbidity when INSURE method (intubation-surfactant treatment-extubation) was used for treatment of very premature infants with RDS. Taken together, there is increasing evidence suggesting intubation and prophylactic administration of surfactant in extremely premature infants.

There are many pathophysiologic reasons why prophylactic surfactant therapy might be more effective and lead to less lung injury than late surfactant therapy for established RDS. In a surfactant-deficient lung of an air-breathing animal, there is ongoing leakage of serum proteins into alveolar space. These proteins act as inhibitors of surfactant function [21]. Jobe et al. [22] and Ikegami et al. [23] have demostrated that early exogenous surfactant administration can reduce the protein leak, and others have shown that early administration of surfactant can lower severe lung injuries in RDS [24, 25].

## 6. Conclusion

From our data we conclude that infants under 27 weeks' gestation are “at risk” in developing RDS and profit from prophylactic surfactant administration by reducing the time of mechanical ventilation. This in turn could contribute to reduce the risk for mechanical ventilation associated complications (interstitial emphysema, pneumothoraces, and chronic lung disease) without any detrimental short-term side effects. As shown in the COIN trial [17], initial nasal CPAP did not significantly reduce the rate of death or bronchopulmonary dysplasia, as compared with initial intubation, but could increase the mortality rate in extremely preterm infants. Therefore, we consider that it is safer to intubate these smallest babies and administer prophylactic surfactant.

Due to the retrospective nature and the small sample size of our clinical trial more prospective, randomized and controlled trials (like the forthcoming report on the NICHD Neonatal Research Network SUPPORT trial [26] and the CURPAP study [27]) are needed to test whether prophylactic surfactant administration in very preterm infants is superior over the selective procedure.

## Figures and Tables

**Table 1:** Patients' characteristics.

	Prophylactic surfactant group (observational period) *n* = 31	Selective surfactant group (historical control) *n* = 34	Significance
Birthwight (g)	709 ± 164	701 ± 159	n.s.
Gestational age (wks+d)	25 + 1 (23 + 1 to 26 + 6)	25 + 3 (23 + 1 to 26 + 5)	n.s.
Male sex (%)	54	41	n.s.
Antenatal steroids (%)	90	91	n.s.
APGAR-Score (5 minutes)	7	7	n.s.
SGA (%)	29	15	n.s.
Multiple births (%)	7	9	n.s.
GA < 24 wks (%)	19	18	n.s.

SGA:small for gestational age (birth weights below 10th percentile); GA:gestational age; wks, weeks; d, days.

**Table 2:** Mortality and morbidity.

	Prophylactic surfactant group (observational period) *n* = 31	selective surfactant group (historical control) *n* = 34	Significance
Death until term	5 (16%)	6 (18%)	n.s.
IVH> II	4 (13%)	3 (9%)	n.s.
PVL	3 (10%)	1 (3%)	n.s.
Pneumothorax	1 (3%)	4 (9%)	n.s.
CLD	8 (26%)	13 (38%)	n.s.
PIE	0 (0%)	3 (9%)	n.s.
Need for mechanical ventilation (h per patient)	74 ± 26	171 ± 35	*P* = .03
Diagnosis of PDA	22 (71%)	28 (82%)	n.s.
Treatment of PDA	21 (68%)	26 (68%)	n.s.
NEC	1 (3%)	5 (15%)	n.s.
Nosocomial infection	14 (45%)	15 (44%)	n.s.
Postnatal steroids	19 (61%)	21 (62%)	n.s.
Surfactant doses	41	62	n.s.
Surfactant doses per patient	1.3	1.8	n.s.

IVH:intraventricular hemorrhage; PVL:periventricular leukomalacia; CLD:chronic lung disease (need of additional oxygen after 36 weeks gestational age); PIE:pulmonary interstitial emphysema; PDA:patient ductus arteriosus; NEC:necrotizing enterocolitis (stadium II or III according to Bell).
